# “Demanding, but Worth It”: The Parental Experience of Home-Based Vojta Therapy for Children Presenting Global Developmental Delay—A Qualitative Study Using Photo-Elicitation

**DOI:** 10.3390/jcm15010045

**Published:** 2025-12-21

**Authors:** Ana San-Martín-Gómez, Roberto Cano-de-la-Cuerda, Carmen Jiménez-Antona, Livia Gomes Viana-Meireles, María Salcedo-Perez-Juana, Jorge Pérez-Corrales, Domingo Palacios-Ceña

**Affiliations:** 1 Research Group of Humanities and Qualitative Research in Health Science (Hum & QRinHS), Department of Physical Therapy, Occupational Therapy, Physical Medicine and Rehabilitation, Universidad Rey Juan Carlos, 28922 Madrid, Spain; ana.sanmartin@urjc.es (A.S.-M.-G.); maria.perezjuana@urjc.es (M.S.-P.-J.); jorge.perez@urjc.es (J.P.-C.); domingo.palacios@urjc.es (D.P.-C.); 2 Research Group Laboratory of Movement Analysis, Biomechanics, Ergonomics and Motor Control (LAMBECOM), Department of Physical Therapy, Occupational Therapy, Physical Medicine and Rehabilitation, Universidad Rey Juan Carlos, 28922 Madrid, Spain; 3 Instituto de Educação Física e Esportes, Universidade Federal do Ceará, Ceará 60455-760, Brazil; liviagviana@ufc.br

**Keywords:** home-based, developmental delay, emotion, Vojta therapy, parents, caregivers-involvement, qualitative research, photo-elicitation, children disabilities

## Abstract

**Background/Objectives:** Introducing a demanding home-based program (HBP) of Vojta therapy (VT) into their daily activities is a life-altering event for parents of children with global developmental delay (GDD). This study aims to document the experiences of parents of children with GDD administering a HBP of VT. **Methods:** A multicentre study with a qualitative case design based on an interpretative approach is presented. Interviews were conducted with 10 parents using photo-elicitation (PE). Inductive and thematic analyses were used. **Results:** Four common experiential themes were identified. Firstly, crying was identified as the most significant barrier to administering therapy (despite parents accepting that crying was not pain-related). Secondly, parents described the intense and variable emotional impact of being responsible for the therapy and its effects on their child. Thirdly, parents unanimously felt that their involvement was worthwhile, with the developmental results they perceived outweighing the emotional, physical and time demands of administering the VT. Finally, parents developed a narrative of hope stemming from the therapy and its observed effects. **Conclusions:** The physical, emotional and time demands on parents when administering a HBP of VT are very significant. The main barriers to adherence to the program are identified as the child’s crying during therapy and time management. Intense emotional experiences, both positive and negative, arise while administering a HBP of VT. Parents are not only able to overcome all emotional and logistical challenges when they recognize improvements in their child, but they also begin to hope for further improvement. Implications for the professional design of HBPs of VT include the following: advanced warning that crying is normal, part of the therapy and not pain-related; training and ongoing support from a qualified therapist; training in recognising developmental improvement; and psychological support to deal with the emotional journey.

## 1. Introduction

Global Developmental Delay (GDD) is a neurodevelopmental disorder characterized by delayed acquisition of two or more developmental milestones [1]. Early intervention is key to improving prognoses for children with GDD [2,3] and motor function improvements are reported to be associated with increased frequency of therapy [4,5,6].

Given the fundamental importance of frequency of therapy sessions, parental involvement in administering therapy in the home is needed to optimise outcomes [7]. This can be achieved using Home-Based Programs (HBPs) [5] in which the child performs therapeutic activities with parental assistance in the home environment [8]. They offer improved frequency of therapy which enhances the children’s development as well as facilitating retention of the improvements [9]. To ensure effective administration of HBPs, parents must be trained by and have ongoing access to the support and coaching of a qualified therapist [10,11]

Given the fundamental importance of frequency and consistency of its application, Vojta therapy (VT) is an ideal candidate for home-based delivery. Its effects have been studied across a wide age range, from newborns to adults [12] as well as in subjects with different neurological disorders, including developmental delay [13,14]. VT is a neuromodulative treatment based on reflex locomotion principles that aims to promote typical innate patterns [15]. It involves the application of pressure to specific stimulation points to activate global innate locomotive patterns or complexes, provoking coordinated, rhythmic activation of the skeletal musculature throughout the entire body [16], thereby improving motor function in individuals with neurological or developmental disorders. VT has been shown to be especially effective in infants and children with motor development delays [13,14,17]. It is designed to harness and promote neuroplasticity by providing structured sensory input that facilitates the reorganization of motor pathways [12].

Since VT stimulation must be repeated from three to four times a day, there is a practical need for parental participation [13,18,19,20,21,22].

However, parents may suffer significant levels of anxiety and stress while performing therapy [21,22,23]. The impact on parents of administering HBPs is well documented for parents of children born prematurely or with cerebral palsy [24,25,26]. Anxiety, stress, child discomfort during therapy, emotional volatility, fatigue or a self-demanding program are some of the barriers encountered by parents when carrying out HBPs [21,22,23,25,27,28,29,30,31]. Nonetheless, there is a lack of comparable information regarding parents of children with GDD.

The current study aims to fill this gap by using PE-based interviews to document the experiences of parents of children with GDD administering VT via HBP.

## 2. Materials and Methods

### 2.1. Design

A case-based qualitative study was conducted, using an interpretive framework [32]. A case-based qualitative study is well-suited to describing the experiences of professionals, patients, and families in the developmental medicine field and in child neurology [33,34] and to understanding the level of acceptance or rejection of treatments.

Seven researchers participated in this study (4 women), including a researcher nurse (DPC), four physical therapists, all of whom are child neurology experts (RCDC, CJA, ASMG, MSP), an occupational therapist (JPC) and a psychologist (LGVM). All researchers work at the University [BLINDED]. Background context of the application of VT in Spain is described in Supplementary Material S1 [13,15,16,19,20].

### 2.2. Participants, Sampling Strategies and Recruitment Procedure

**Figure 1:**
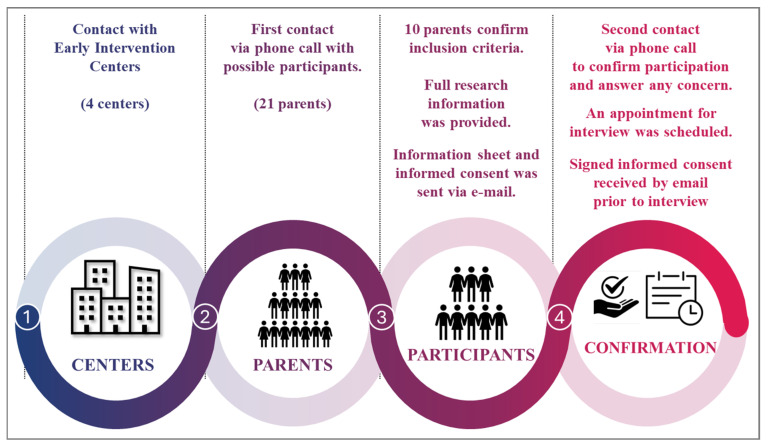
Participant recruitment procedure.

Purposeful sampling was used [35]. Parents of GDD children already being treated with VT were recruited from four Early Intervention Centers (EICs). Potential participants were contacted through the EIC’s physiotherapists. See Figure 1.

To be included in this study, parents needed to fulfil the following inclusion criteria: (a) be the parent of a child diagnosed with GDD (without an identified metabolic, genetic, or neurological cause) by a neurologist (code 315.8 (F88) DSM-V); (b) be receiving training in VT from a trained professional with a recognized certification from the Spanish Vojta Association (www.vojta.es, accessed on 1 September 2023); and (c) to have been administering VT to their GDD child at home more than 3 times a week for at least for 2 months before being recruited for the study. Exclusion criteria were parents of GDD children who perform VT ≤ 3 times a week.

There are different proposals for estimating valid sample size in qualitative research (pragmatic considerations, saturation, power of information, etc.) [36] as there is no single, authoritative formula for calculating it [35]. Previous studies using empirical tests determined the number of interviews necessary to achieve saturation [37,38]. A saturation proposal by Hennink & Kaiser [38] suggests that data saturation is achieved by interviewing between 9 and 17 participants in qualitative studies where the objectives of the study are narrowly defined and the populations under study are homogeneous. With this proposal, a greater capacity to identify codes, categories, and topics is achieved. In addition, the current proposal also helps researchers to know a reference number to stop collecting data and/or recruiting participants. Previous qualitative studies into the experiences of parents of children with disability or neurodevelopmental disorders using photo-elicitation used sample sizes of between 10 and 15 parents [39,40]. Given the limited size of the target population for this study, all available cases were included to obtain a greater richness of the data.

### 2.3. Data Collection and Procedures

This study used photo-elicitation (PE), a technique of research collaboration where participants submit photographs they feel are representative of their experiences [41,42] and discuss their meaning with the researcher [43,44,45]. This technique allows participants complete freedom to establish what is important to them [46], thereby helping the researcher to understand the full breadth of participants’ experiences (including emotions, feelings and ideas) [43,44,45] without imposing limitations on discussions via a pre-constructed question framework [46].

**Figure 2:**
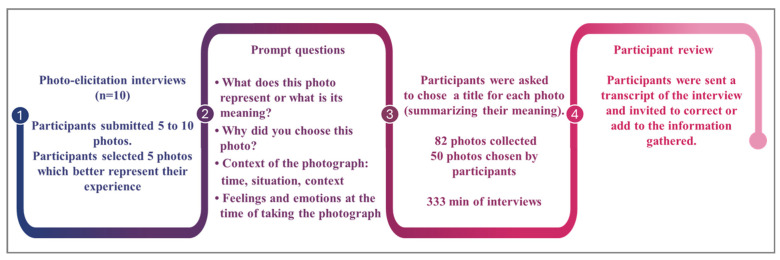
Data collection procedure.

Participants received guidelines for taking photographs to ensure confidentiality (e.g., prohibiting photographs of faces or other identifiable information). They selected photographs to send to researcher ASMG, and dates were set for interviews with researchers ASMG, CJA and LGVM. In the interviews, each participant selected five photos that they felt best represented the application of VT at home. The researchers collaboratively developed four prompt questions for the photo-elicitation interviews, following recommendations for the use of photo-elicitation as a data collection method in qualitative research [41,43,46]. These questions were open-ended and enabled the researchers to explore key aspects related to the implementation of VT within the HBP modality. Additionally, participants were asked to create a title for each photo, to summarize its meaning to them. A total of 82 photographs were submitted, of which 50 photographs were chosen by participants as those best representing VT in the home. See Figure 2 for the data collection procedure.

The interviews were conducted via a private video chat room using the Microsoft Teams platform (https://www.microsoft.com/es-es/microsoft-teams/log-in, accessed on 1 September 2023). Each participant received a private and personalized email with an invitation. All interviews were conducted by three researchers (ASMG, CJA and LGVM). Additionally, researcher field notes were used as a secondary source of information. Subsequently, interview transcripts were sent to all participants who were asked for their approval of the content and whether they wanted to make corrections or add any further information.

### 2.4. Data Analysis

**Figure 3:**
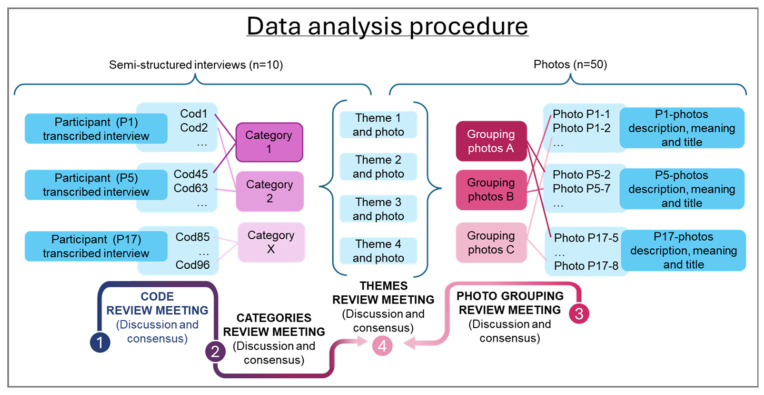
Data analysis procedure.

All interviews were recorded, and transcripts were submitted to inductive thematic analysis [34] which generated codes that were reviewed by the researchers. The researchers then developed categories from these codes and, following discussion, reached consensus on themes. Also, to confirm, triangulate, deepen and enrich findings, an analysis of narratives related to the photographs was made, with photos being grouped based on their title, description and meaning. Further discussion was held around categories and groupings to identify possible themes. Figure 3 depicts this data analysis procedure. The analysis was conducted by two researchers (ASMG, CJA) with expertise in qualitative analysis in health sciences and specialized knowledge of VT. The researchers performed double independent coding of all interviews.

Throughout the analysis process, matrices were developed using Microsoft Excel, to better understand the relation between emerging themes and participants’ images [34]. A further interdisciplinary meeting of the researchers was held in which consensus was reached on four final themes. During team meetings, researchers from diverse professional backgrounds (nursing, occupational therapy, psychology, and pediatric physiotherapy) contributed insights and comments informed by their respective areas of expertise. Suggestions from nursing focused on the implementation of care within the HBP modality; occupational therapy emphasized the recovery of meaningful occupational activities; the psychologist addressed concerns related to the emotional well-being of parents, whereas physiotherapy concentrated on the rehabilitation process and the application of VT in HBP. Each theme was linked to specific quotations and the corresponding photographs. No qualitative software or Artificial Intelligence was used. Furthermore, descriptive statistics were used to calculate the central tendency (mean) and dispersion (standard deviation) for continuous variables; and frequencies and percentages for categorical variables of the participants’ sociodemographic data. These analyses did not involve statistical inference and were used to describe and summarise the characteristics of the data. In qualitative research, the purpose of collecting these data is to provide information that will enable a more comprehensive contextualization of the participants’ results [47].

### 2.5. Rigor

The procedures used to control trustworthiness are described in Table 1 [48,49]. Also, the Standards for Reporting Qualitative Research (SRQR) [50] and the Consolidated Criteria for Reporting Qualitative Research (COREQ) were followed [51].

### 2.6. Ethics

This study adhered to the principles of the Declaration of Helsinki and received ethical approval from Ethic Committee of Universidad [BLINDED] (code: 3006202326423). All participants provided written informed consent: (a) to be included in the study; (b) for the researchers to publish or otherwise disseminate narratives obtained in the study; (c) for the researchers to use photographs in the public domain. Written informed consent was collected before enrolment in the study and data collection. Recorded video-audio interviews and participants’ photographs were anonymized via an alphanumeric code.

## 3. Results

Ten parents of GDD children were recruited (3 fathers). Their mean age was 42.40 years (SD 4.93). Four themes were identified, namely (a) crying during therapy, which was the principal demotivator for parents; (b) parents experienced an “emotional rollercoaster”; (c) parents saw their efforts as worthwhile; and (d) the program gave parents hope. Table 2 shows the participants’ demographic information and their children’s ages.

Narratives that justify and confirm the traceability of the results [49] are shown in Table 3, Table 4, Table 5 and Table 6.

### 3.1. Crying During Therapy

Participants universally acknowledged that their child’s crying while they administered VT represented the biggest challenge. They frequently used phrases such as “s/he cries a lot” in reference to their child’s reaction during therapy. Crying was identified as an additional, highly emotional effort that parents had to make for their children every time they performed VT. Several participants related that the Vojta therapist explained to them during their first session that their child would cry every time they performed VT but that they were not hurting their child and that the crying would stop the moment they ended the therapy session. Seven participants agreed that their child was not in pain during the VT sessions and observed that crying ceased as soon as VT stopped. Two of the remaining three participants, pointed out that even if there was no pain, there was certainly some discomfort. All parents thought the crying sounded more like a protest than a reaction to pain, concluding that it was their child’s way of communicating that they did not like the positions and effort required of them.

Participant PE9 took a photo of the door of the bedroom where VT was performed to illustrate his need to close the door before therapy sessions because of his child’s crying.

Another participant experienced relief when his child also cried in VT sessions at the hospital, noting that this was a normal reaction to the therapy and not as a result of him doing something wrong.

All parents reported that their child’s persistent crying was the greatest difficulty they faced while performing VT at home, despite them fully understanding that their child was not in pain.

### 3.2. Emotional Rollercoaster

This theme encompasses the volatility and strength of emotions experienced by parents while performing VT with their children, a theme that all participants referred to. The emotional complexity experienced by the parents at the onset of HBP is described as distressed by crying, guilt, moments of tension that, over time, transform into focus and unwavering self-discipline to find the strength needed to complete the exercises. They accept tiredness and exhaustion with resignation as they are aware of the results which instill hope in them, a mix of emotions that can vary even within the same day. Two participants clearly identified this concept through the titles of their photographs. One titled a photo “emotional rollercoaster” (PE8) to represent his volatile relationship with VT, while another chose “emotional cyclogenesis” (PE5) as a metaphor to describe the sudden onset of intense emotional states, comparable to a storm of emotions.

Participants described their emotions as conflicting, ranging from heartbreak at the apparent discomfort and crying of their child to elation when they observed progress and hope about the future goals, they could help their child attain. Some participants referred to the emotional experience as one of “light and shadows,” with hope and discouragement sometimes present in equal measure. Others saw the program as a true fight between head and heart.

This conflict was starkly observed in the photographs. Two participants (PE1, PE5) photographed their hands to represent their relationship with VT. However, one said the photo represented the hands that were guilty of causing discomfort to his son while the other said it represented her “healing hands” which help her son improve. Participant PE2 expressed this emotional conflict through two photographs, one depicting a sunset over a calm sea while the other was of Edvard Münch’s painting “The Scream”. She described how The Scream represented the emotionally and physically exhausting effort of performing VT on her daughter at home while the calm sea represented her joy and peace of mind as her daughter achieved new developmental milestones.

Participants linked the administration of VT to negative emotions, using adjectives such as sorrow, sadness, discomfort and self-suffering. Repeated performance of VT, several times a day, every day of the week, also provoked a constant feeling of obligation, described as a “tick-tock in the head of self-demand” (PE3). For participants, skipping a VT session, for whatever reason, represented a worsening of their children’s condition, a delay in their development and improvement. This increased stress, with participants not allowing themselves to feel unwell because therapy had to be done, relegating their own well-being to the background in order to “pluck up [the] courage” (PE5) to perform VT. Participants with more than one child also felt guilt as performing VT meant spending less time with their developmentally normal child(ren).

Perceived outcomes from VT determined parents’ emotional balance. If they perceived positive outcomes, therapy was described as “wonderful”, if not, they doubted whether the effort was worth it. Some participants (PE1, PE2, PE4, PE6, PE7) described how new milestone acquisition encouraged them to continue with VT. For three participants (PE1, PE6, PE7), performing VT instilled feelings of calm and hope, as they observed positive results in their children from the first session.

All participants perceived VT positively as they observed their child’s improvement, despite some having concerns about the discomfort they caused their child. VT was regarded as a necessary tool, a costly path to achieve a happy ending.

Half of the participants had tried various alternative therapies and had felt frustrated at not seeing any improvement in their children. VT provided them not only with improvements but also with the information they needed to understand the process, “VT hit the nail on the head” (PE7). This helped them develop patience and understand that they needed to work and make the effort.

### 3.3. The Effort Is Worthwhile

This theme was shared by all participants who, while recognising the very significant demands placed upon them, felt that the results were certainly worth it.

Compared to conventional treatment, where parents take their children for a weekly session at an EIC, the performance of therapy in the home, by parents, several times a day, every day of the week, represents a radical change, one that entails a very significant commitment by parents. The participants, eager to do everything in their power to assist their child’s development, both recognised this and assumed the obligations from the outset. In the words of PE9, “it’s what we have to do”. The practical impact on their lives of this commitment, as perceived by the participants themselves, are described below.

Learning to perform VT requires effort and total concentration: “Well, it is not as easy as it seems! Then you must do it at home. If you don’t know how to do it, you are lost.” (PE10). Participants also noted that administering VT during vacations away from the home was even more stressful: “we struggle to perform VT out of the home environment, it is an effort, it is not as natural, you lose your routine” (PE3).

The sheer amount of time invested by parents in performing VT several times a day was another commonly raised issue, with some participants opting to wake up earlier to accommodate the therapy along with all the other demands on their time. Some participants submitted photos of watches to represent this.

Some parents found the physical effort of administering VT exhausting: “Prepare yourself for VT because it is not easy at all. It is extremely hard work which exhausted me” (PE2). Participants were also aware of the effort put in by their child with some being astonished by their children’s stamina and the amount they perspired: “I realize how much effort it requires. When he gets up and you see all the sweat he produces on the stretcher and I see him sweating, I realize how much effort it takes and how willing my son is to exercise.” (PE4). Parents reported feelings of pride at seeing this level of effort from their child, and the resulting improvements. Children effort during therapy and their daily improvement made parent proud. Participant PE7 represented her daughter daily effort in a photograph, through a stairs image: “I look at my daughter with pride. She is working, even if she does not like it. Her effort is leading her to grow, to move more and better, to improve little by little every day. She makes me proud” (PE7-Photo4).

It is worth noting that half of the participants shared the task of performing VT with their partners. The parents described how at the onset of HBP, they preferred to do exercises together for mutual support and encouragement. With experience, several couples then opted for individual practice, as their children were less distracted, simplifying organization at home. Working together allowed each parent to do different exercises. The sense of collaboration in supporting their child was a recurring theme among couples, who were grateful to be able to share this additional responsibility with their partner. Apart from spreading the workload, this created a positive “team vision” (PE3) as well as allowing the immediate sharing of concerns and joint celebration of improvements.

Notwithstanding the emotional, physical and time demands on parents administering VT in the home, perceived outcomes were universally deemed to be worth it: “Even though the journey is difficult, in the end, you reap the fruits of your labour” (PE5). Observe outcomes has been properly seated with his sister on the beach (PE1), an open hand to manipulate and play (PE2), been able to lift his legs from ground (PE3) or start walking (PE7) encouraged them to continue. Participant PE6 represented an arrow on the ground in a photograph to express her progress with VT.

Some participants perceived improvements right from the first session or reported new milestones being reached within just a few sessions: “On the first day of therapy, the child cried a lot. But as he was on his tummy, the improvement became evident immediately. He stood up much more. He had a hard time, but it was worth it” (PE9). This early perception of progress is helpful in ensuring adherence to the program.

### 3.4. Hope

The word “hope” arose frequently in interviews and specifically in relation to seven participants’ photos: “[VT] has given us hope, because we have seen its effectiveness. It is the hope that someone gives you when they say, “come on, I will help you”” (PE7). Hope was associated with gratitude, optimism and calmness about the achievements made through VT.

Participant PE5 retrieved an old photograph, from her personal album for the study, because when she stopped to think about what VT meant to her, she discovered a feeling of reward, of hope for the future, and that photo came to mind her, to represent the future, a happy ending she longed for with VT, a reward obtained for having helped her son achieve a better future. Another participant (PE6) submitted a photo of a painting of green leaves which to her represented the (green) hope she had placed in VT.

All participants expressed a desire for a better future, a longing or yearning to achieve this through VT. Participant PE8 represented this yearning in a photograph which showed the window of the room where he performed VT. The window looked out on to a school playing field. Through this window, he “saw” a future in which his son was playing football and not lying on the treatment table.” We are inside the room where we do therapy, this is the window of that room, outside there is the school, where normal everyday life takes place, which is what we want, for my son to be able to run, jump, have a job….That window is our goal for the future.” (PE8)

The future was present in all interviews, with participants hoping for a time when their children reach the developmental milestones that correspond to their age. Participant PE5 represented the future through an image of a child’s milestone chart to show the progress he hoped for. Another participant aspired to a future where developmental delay was overcome, and VT would be no longer a part of their lives. She titled her photo “This shall pass” (PE3). Other participants spoke of their desire for their child to have a normal upbringing and toys rather than an exercise mat in their bedroom.

## 4. Discussion

This present study describes the experiences of parents of children with GDD undergoing Vojta therapy in a HBP, highlighting crying as the main barrier to treatment, the variability of emotions they face, and how despite these situations, the effort is worthwhile, with all parents sharing a discourse of hope.

Parental implication in home delivery therapy is fundamental. However, the high levels of anxiety and stress that are present while performing therapy [21,22,27] may lead to non-adherence to a HBP [23,28,29,30]. Advance knowledge of the main factors likely to cause parents to consider abandoning a HBP enables better education and more appropriate management of parental needs, thereby increasing the probability of adherence to and the eventual success of the HBP.

The factors that undermine adherence to a HBP for children with GDD have been widely studied, with the perception of the child’s discomfort during therapy being identified as a key detractor [28,29,52]. Strojek & Wójtowicz [18] pointed out how VT performance compels the child to adopt positions that cause discomfort and trigger crying. As stated by Gustafsson & Levréro [53], crying is the infant’s broader means of communication, and through its intraindividual variation, it can communicate distress, pain, or other needs and parents are able to recognize these variations, thus enabling them to provide adapted care. Therefore, crying could be identified not as a reaction to pain [12,18], but with protest at the discomfort caused by VT. All participants in the present study reaffirmed this, noting the “protest” nature of their children’s crying. Previous studies highlighted how the prior education of parents by professionals helped maintain the effectiveness and adherence to treatment at home [26,52,54,55]. Pre-therapy information about the onset of and reasons for crying, and how to manage the emotions it provokes, are strategies that should be considered when prescribing HBP with VT.

Our participants described suffering significant emotional volatility with pronounced highs and lows, the highs being directly linked to observed results. Previous studies on parents administering therapy to children born prematurely or with cerebral palsy indicated the relation between emotional fluctuation and therapy outcomes, with parents adhering to HBP if improvements are perceived, or conversely becoming demotivated and abandoning it in the absence of perceived improvements [25,31]. Strojek & Wójtowicz [18] explained how parents who perform VT at home experience emotions arising from overburdening and stress, although also report moments of highly rewarding personal growth. Previous studies have shown how constant caregiving diminishes parents’ quality of life and well-being and increases their stress [23,30,56]. Parents become extremely self-demanding and suffer guilt when they are unable to perform the treatment at home due to fatigue or other commitments [23,30,56]. Therefore, in the design of HBP with VT, special consideration must be given to the emotional support of the parents.

In contrast to these negative impacts, previous studies have also indicated how parents’ involvement empowered them, giving them a strong sense of purpose [18,30,56,57]. They also experienced personal growth [58] and an appreciation of their children’s strength and determination [25,27,59]. This is aligned with the findings of this study, in which all 10 participants remained committed to the HBP throughout. While recognising the significant physical, emotional and diary challenges, they were grateful to be able to contribute and proud of the efforts and progress of their children.

This study also identified hope as an important motivational factor for parents, encouraging them to continue participating in their children’s therapy. This hope is generated by their perception of the effectiveness of VT. Two previous studies on VT related how parents with children with developmental disorders wanted to participate in building and fighting for their children’s future, they hoped for a better future for them [18,27]. Ochandorena-Acha & Noell-Boix [25] went on to describe how parents longed for a time when they could relax at home without therapy, a desire shared by some participants of this study.

### 4.1. Limitations and Strengths

This study has limitations in terms of generalizability, as results of qualitative study, cannot be extrapolated to all parents of children with GDD administering VT via HBP. However, our findings could be applied or prove useful in other contexts to understand the behaviors and actions of other groups experiencing similar circumstances (transferability) [49], and to assist rehabilitation professionals in comprehending the implications of HBP with VT. Secondly, participants in this study were recruited based on their ability to provide information relevant to addressing the research question or objective, owing to their experience of being parent of a child with GDD [32,35]. Other variables or social, educational, cultural, or economic factors that might influence their perspective on implementing a treatment at home were not considered during recruitment. Future research should take these factors into account to examine diverse participant profiles with varying economic and educational characteristics. Third, participants were recruited exclusively from centers where HBP with VT was administrated. This could bias the results toward a positive view of the treatment. However, participant selection in qualitative methodology is aimed at obtaining relevant information to answer the proposed study objective (non-probability sampling) [35,48]. In qualitative research, positive bias may arise from researchers’ expectations when questions are framed to elicit specific responses or when participants seek to please the researchers. As a result, data may be interpreted and presented in a more favorable manner [60]. To minimize this bias, several strategies are recommended: (a) triangulation (use of multiple data sources and methods); (b) reflexivity, whereby researchers acknowledge and reflect on their influence on the research process; (c) the use of open, neutral, and non-directive questions; and (d) member checking [61]. These strategies were applied in the present study. In addition, interviews were conducted by two researchers with experience in VT and a third researcher, a psychologist, with no prior experience in VT.

In our knowledge this is the first study to describe the experience of parents of children with GDD administering a HBP of VT. Qualitative research, helps to understand the use of therapies, due to its capacity to explore meanings, sociocultural contexts, and dynamics among professionals, patients, and families [62,63]. Thus, qualitative research allows us to study how practices and discourses are constructed around different therapies and to describe the beliefs, values, and subjective experiences of patients and professionals who choose these therapies [62,63]. A photo-elicitation methodology was chosen for data collection which allows for triangulation of the data and the incorporation of information from different data sources [49] to build a more complete picture. The wide range of findings reflect the use of this more open research technique. Furthermore, the findings are derived exclusively from the photographs taken and selected by the participants. An inclusion and analysis of the non-selected photographs could potentially yield additional insights into the aspects of their experience that parents chose not to elaborate on or deemed too private to disclose.

### 4.2. Implications for Practice

Rehabilitation professionals should focus on minimizing and mitigating the negative impacts on parents (e.g., through pre-therapy education and technical and emotional training and support) and on ensuring that parents are adept at perceiving improvements.

The detailed findings of this study reveal some consideration when prescribing HBP-delivery of VT. These include providing advance notice of the onset, persistence and meaning of crying, helping to manage the time demands, and recognizing the effort that parents make with their commitment. We recommend that therapists collaborate with parents to co-design personalized and flexible therapy schedules (e.g., adaptable timing, integrating exercises into daily routines) rather than insisting on a rigid ‘three-times-daily’ structure. In addition, developing educational programs aimed at addressing the implications for parents undertaking HBP’s with VT are essential.

## 5. Conclusions

Home delivery of VT is physically and emotionally demanding on parents. Their children’s crying during therapy is the most significant barrier to continuance, followed by the large and inflexible time commitment.

One novel aspect of our results is the “emotional rollercoaster” parents experience while performing HBP with VT but are able to overcome this as they recognize improvements in their child and conclude that their significant commitment is not only worthwhile but a source of real hope for the future.

The key experience from this study is that the developmental results achieved through HBP-delivery of VT are sufficient to ensure parents’ adherence to the program.

Furthermore, future new lines of qualitative research could be developed to further explore the emotional dimension of therapeutic experiences implemented in the family context with other therapies.

## Figures and Tables

**Table 1:** Trustworthiness criteria.

Criteria	Techniques Performed
Credibility	Researcher triangulation: each interview was analysed by two researchers. Subsequently, interdisciplinary team meetings were held in which the analyses were compared and themes identified.
	Triangulation of data collection methods: information from PE interviews was compared with researchers’ field notes.
	Participant checking: all participants were offered the opportunity to review the transcripts to confirm their content. None of the participants made any corrections or additional comments.
Transferability	In-depth descriptions of the study were made, detailing the characteristics of the researchers, participants, sampling strategies and data collection and analysis procedures.
Dependability	Audit by an external researcher: an external researcher evaluated the study’s research protocol, focusing on aspects related to the methods applied and the study design.
Confirmability	Researcher and data collection triangulation: Researcher reflexivity was encouraged through reflective reporting.

**Table 2:** Participants’ demographic information and children’s age profile.

Participants	10 Participants (3 Fathers, 7 Mothers)
Age of participants	Mean: 42.40 years (SD 4.93)
Work situation	Active: *n* = 5 Leave of absence: *n* = 3 Parental leave: *n* = 2
Number of children	Only child: *n* = 5 More than one child: *n* = 5
Educational status	College education: *n* = 10
Time in Vojta Therapy	Between 2 and 3 months: *n* = 5 Between 3 months and 1 year: *n* = 5
Child’s age	Less than 6 months old: *n* = 2 More than 6 months and less than a year old: *n* = 5 Older than a year: *n* = 3

SD: Standard deviation.

**Table 3:** Narratives of theme 1: Crying during therapy.

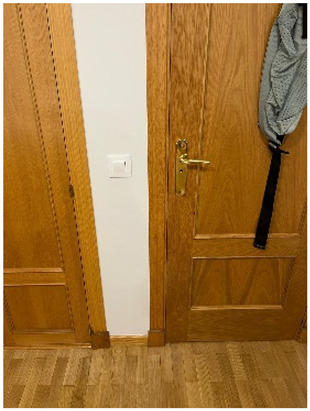
*Title: “Prevent crying from being heard” PE9-Photo5* *“We close the door to perform the exercise because….well, ….he cries a lot” (PE9)* *“It was a cry of (makes crying sound) like a protest, it was a cry of «leave me alone», it was not a loud cry” (PE3)* *“He cries….as you force him to do a certain movement that is difficult for him, so that he gets better...heals” (PE5)* *“Yes, yes, the guy fights, he fights it, because he doesn’t like it, well, he cries as much as he can” (PE6)* *“Well, it bothers him, because... like everyone when we go to the gym, it bothers us, you see, nobody likes it. I understand him. It’s like going to the gym to do 500 sit-ups. When you’ve already done 100, you say, «look, screw it, I’m going home to have a coffee»“ (PE7).* *“Yes, it stops, it stops right away, I mean, right away… a few seconds after you stop doing it, [he] stops [crying]” (PE8)* *“The child cries a lot. It is true that we know that he only cries during the exercise; when you release him, he stops crying” (PE9)* *“Look, it is supposed to be normal for it not to hurt, he has to feel uncomfortable to achieve results, but it doesn’t have to hurt” (PE10)*

**Table 4:** Narratives of theme 2: Emotional rollercoaster.

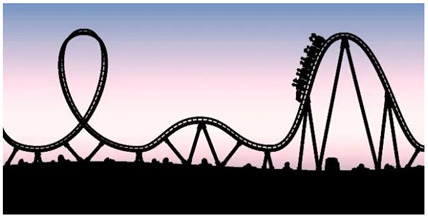
*Title: “Emotional roller coaster” PE8-Photo 5* *“Well, it’s an emotional roller coaster, with therapy, with its ups and downs. With its moments of hope and its moments of discouragement. It depends a lot on the results you get, because sometimes it’s rewarding, and other times you think it’s not worth much.” (PE8)* *“Emotional cyclogenesis, is like a contradiction between ideas and emotions, right? You get a lump in your throat when you go there. You know it is going to be a bad time, but you go because you know it is going to do good. It is contradictory” (PE5)* *“Well, I have a bad time (lowers her tone), I don’t like seeing her cry, I DON’T LIKE seeing her cry. But I accept that it’s something that’s good for my daughter and that it must be that way.” (PE10)* *“Being exhausted in the morning and then seeing her reach a new milestone, makes me want to keep fighting” (PE2)* *“VT hit the nail on the head” (PE7)*

**Table 5:** Narratives of theme 3: The effort is worthwhile.

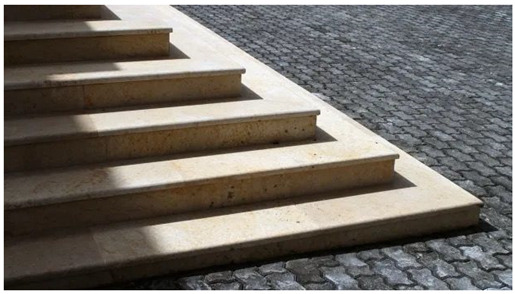
*Title: “The effort of everyday life” PE7-Photo4* *“I look at my daughter with pride. She is working, even if she doesn’t like it. Her effort is leading her to grow, to move more and better, to improve little by little every day. She makes me proud” (PE7-Photo4).* *“It represents my daughter’s effort, and also the effort we make as a family. But above all, it represents the girl’s, who at the end is the one who has shown us this. With help, with the right help, she has managed to walk, and she’s progressing, and quite quickly, according to what the physiotherapists tell me.” (PE7)* *“Well (Name of his son)’s work, perseverance, dedication, effort, and discipline every day.” (PE8).* *“For me, this represents progress. I’m seeing that the child is making progress in the work we’re doing. We’re achieving significant improvements. The photo was a way of representing the progress.” (PE6)* *“We struggle to perform VT outside the home environment, it is an effort, it is not as natural, you lose your routine” (PE3)* *“Prepare yourself for VT, because it is not easy at all. It is extremely hard work which exhausted me” (PE2)* *“I realize how much effort it requires. When he gets up and you see all the sweat he produces on the stretcher and I see him sweating, I realize how much effort it takes and how willing my son is to exercise.” (PE4).* *“Well, it is not as easy as it seems! then you must do it at home. If you don’t know how to do it, you are lost” (PE10)* *“Even though the journey is difficult, in the end, you reap the fruits of your labour” (PE5)* *“On the first day of therapy, the child cried a lot. But as he was on his tummy, the improvement became evident immediately. He stood up much more. He had a hard time, but it was worth it” (PE9)*

**Table 6:** Narratives of theme 4: Hope.

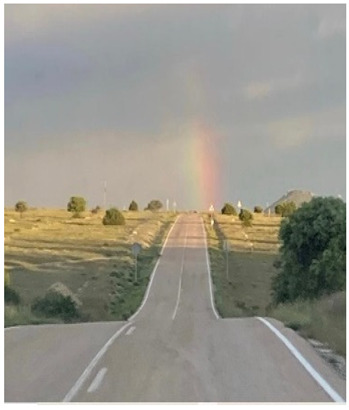
*Title: “The end of the road” PE5-Photo7* *“I took this photo a while ago after finishing a hike, a steep and difficult path, but with a rainbow at the end. The end is worth it! Right? You know? Especially with a view to when I finish the therapy. Even though the path is difficult, in the end, you’ll get your reward. Uh...the fruits of labour.” (PE5-photo7)* *“[VT] has given us hope, because we have seen its effectiveness. It is the hope that someone gives you when they say, -come on, I will help you--“ (PE7)* *“We are inside the room where we do therapy, this is the window of that room, outside there is the school, where normal everyday life takes place, which is what we want, for my son to be able to run, jump, have a job….That window is our goal for the future.” (PE8)*

## Data Availability

The data presented in this study are available on request from the corresponding author due to ethics and legal restrictions.

## Supplementary Materials

The following supporting information can be downloaded at: https://www.mdpi.com/article/10.3390/jcm15010045/s1, Supplementary material S1. Context of Vojta therapy application in Spain.

## Abbreviations

The following abbreviations are used in this manuscript:

EIC	Early Intervention Centers
GDD	Global developmental delay
HBP	Home Based Program
VT	Vojta Therapy
PE	Photo-elicitation
