# Supplementary material for: “Demanding, but Worth It”: The Parental Experience of Home-Based Vojta Therapy for Children Presenting Global Developmental Delay—A Qualitative Study Using Photo-Elicitation
Source: J Clin Med. 2025 Dec 21;15(1):45. doi: 10.3390/jcm15010045 (PMC12786593; doi:10.3390/jcm15010045)
Supplement: Supplementary file 1 [file jcm-15-00045-s001.zip › jcm-4042949-supplementary.pdf]

Supplementary material S1. Context of Vojta therapy application in Spain.

In Spain, training in Vojta Therapy (VT) is regulated by the Spanish Vojta Association (www.vojta.es). This association is responsible for providing training through the Vojta therapy course for children and adolescents, which lasts 320 hours (https://vojta.es/xii-curso-de-terapia-vojta-en-alteraciones-motoras-infantiles-un-exito/).

This training is certified by said association and by the International Vojta Association (www.vojta.com).

The training in VT, through the aforementioned course, is based on two main blocks: on the one hand, the kinesiological assessment, and on the other hand, the treatment.

The assessment involves a theoretical description and practical evaluation of the child's kinesiological development, reflexes, and postural reactions in patients. With this knowledge, the therapist can analyze the child's postural pattern, postural deficits or anomalies, or developmental delays. The second block is based on the theoretical and practical learning of therapy. This section covers the foundations of the therapy and the patterns described by Dr. Vojta, based on defined postures and the activation of specific stimulation points [13, 15, 16, 20]. Applying these points elicits muscular responses that trigger motor patterns. The course also provides training in working with the family, including communication skills, emotional management, positive leadership, listening to parents, and collaborative planning of goals and the therapeutic plan. (https://www.vojta.com/es/formacion-adicional/cursos)

In each therapy session with the family, the therapist assesses the child's motor development and discusses it with the parents. They then jointly establish the goals to be achieved and develop a treatment plan. The therapist explains the correct posture and stimulation areas to the parents. With the therapist's guidance, the parents apply the therapy to their child. The therapist provides guidance in carrying out the therapy and gives guidelines regarding stimulation time and expected responses. It is a continuous learning process, and it is recommended that parents express any doubts or difficulties they encounter with the therapy in each session so that the intervention can be adjusted at home. [15]

The duration of the VT sessions ranges from 5 to 20 minutes, and the ideal dosage is four times a day. Therefore, conducting VT with parents is an intensive therapy based on the principles of neuroplasticity. Families visit the early intervention center once a week to receive counseling and treatment for their child and apply the therapy at home the rest of the time, that is, several times a day. This requires a teaching and learning process for VT, from therapists to parents. [13, 15, 19, 20].
